# Myeloma cast nephropathy with diffuse amyloid casts without systemic amyloidosis: two cases report

**DOI:** 10.1186/s12882-020-02204-x

**Published:** 2021-01-06

**Authors:** Zi-hao Yong, Xiao-juan Yu, Zi-shan Lin, Fu-de Zhou, Xi-nan Cen, Su-xia Wang, Ming-hui Zhao

**Affiliations:** 1 Renal Division, Department of Medicine, Peking University First Hospital, Beijing, 100034 People’s Republic of China; 2 Institute of Nephrology, Peking University, Beijing, 100034 People’s Republic of China; 3 Renal Pathology Center, Institute of Nephrology, Peking University, Beijing, 100034 People’s Republic of China; 4 Key laboratory of Renal Disease, Ministry of Health of China, Beijing, 100034 People’s Republic of China; 5 Key Laboratory of CKD Prevention and Treatment, Ministry of Education of China, Beijing, 100034 People’s Republic of China; 6 Peking University, Beijing, 100871 People’s Republic of China; 7 Peking-Tsinghua Center for Life Sciences, Beijing, People’s Republic of China; 8 Department of Hematology, Peking University First Hospital, Beijing, 100034 People’s Republic of China; 9 Laboratory of Electron Microscopy, Pathological Center, Peking University First Hospital, Beijing, 100034 People’s Republic of China

**Keywords:** Multiple myeloma, Multiple cast nephropathy, Amyloid

## Abstract

**Background:**

Multiple myeloma (MM) is a plasma-cell derived hematologic malignant disease. The malignant proliferating plasma cells secrete massive monoclonal immunoglobulins which lead to various pathologic types of renal injury. Myeloma cast nephropathy (MCN) is the most common histopathologic lesion with the worst renal prognosis. Rarely, the free light chains in the protein casts can form amyloid fibrils. Here, we reported two rare cases of MCN with diffuse amyloid casts.

**Case presentation:**

**Case 1:** A 54-year-old Chinese man presented with a 4-year history of multiple myeloma, proteinuria and hematuria. He had monoclonal IgAλ plus free λ spike in both serum and urine. He had been on chemotherapy for 4 years and maintained normal serum creatinine until 11 months ago. Then, his renal function deteriorated and he went on hemodialysis 4 months before admission. Renal biopsy showed diffuse amyloid casts in the tubular lumens, without any obvious amyloid deposits in other kidney compartments or signs of extra-renal amyloidosis. The amyloid fibrils formed around mononuclear cells which were CD68 negative. According to the morphology and location, these mononuclear cells were considered as tubular epithelial cells. The patient was maintained on chemotherapy and hemodialysis. He died 8 months after renal biopsy.

**Case 2:** A 58-year-old Chinese man presented with a one-and-a-half-year history of proteinuria and slowly rising serum creatinine. He had monoclonal IgDλ spike in both serum and urine. Amyloid casts were observed in the tubular lumens and mononuclear cells could be identified in the center of some casts. There were no amyloid deposits in other kidney compartments and no sign of systemic amyloidosis. The patient also had fine granular deposits along the tubular basement membrane with λ linear staining along tubular basement membrane suggesting light chain deposition disease. He was treated with bortezomib-based chemotherapy followed by lenalidomide-based chemotherapy and achieved very good partial remission (VGPR). After 27 months of follow-up, the patient still showed no signs of systemic amyloidosis.

**Conclusions:**

These 2 cases of MCN with diffuse amyloid casts have different histopathologic characteristics from the usual myeloma casts and tubular epithelial cells might play important roles in the pathogenesis.

## Background

Multiple myeloma (MM) is characterized by plasma cell neoplastic proliferation and overproduction of monoclonal immunoglobulins or free light chains, leading to multiple organs/systems damage. Renal injury is one of the most common complications. Studies have shown that about 40% of patients had serum creatinine above the upper normal limit at diagnosis, and 10% of them required dialysis [1–4]. Renal injury in MM can manifest as different histopathologic types, such as myeloma cast nephropathy (MCN), light-chain amyloidosis, monoclonal Ig deposition disease (MIDD), light chain proximal tubulopathy (LCPT) and tubulointerstitial nephritis (TIN). Among them, MCN is accounted for 90% of renal damages in MM [5]. Rarely, the free light chains in the protein casts can form amyloid fibrils. Here we presented two rare cases of myeloma cast nephropathy with diffuse amyloid casts.

## Case presentation

### Case 1

A 54-year-old Chinese man presented with a 4-year history of multiple myeloma (IgAλ), proteinuria and hematuria. He had been receiving intermittent chemotherapy including TD (thalidomide and dexamethasone), TCD (thalidomide, cyclophosphamide and dexamethasone), PD (bortezomib and dexamethasone), PCD (bortezomib, cyclophosphamide and dexamethasone) and PDDT (bortezomib, doxorubicin, dexamethasone and thalidomide) for 3 years. The hematologic response was not available. He had persistent hematuria and proteinuria with normal serum creatinine (SCr). Then, his SCr increased to 1.64 mg/dl (normal range: 0.50–1.50 mg/dl) 11 months before admission, and 6.18 mg/dl 4 months before admission and went on hemodialysis. One month prior to presentation, he received one course of PTD (bortezomib, thalidomide and dexamethasone) chemotherapy. Then he was referred to our division in September 2017 to further evaluate the renal dysfunction.

He had a 10-month history of hypertension (160/100 mmHg) and was treated with nifedipine and metoprolol. Blood pressure was controlled around 130/80 mmHg. He also had a 50-year history of asthma. His family history was unremarkable. On admission, his medical examination revealed blood pressure 128/78 mmHg, heart rate 79 beats/min and respiration rate 18/min. He was pale, and wheeze was heard in both lungs. Hepatomegaly and splenomegaly were noticed.

After admission, complete blood count showed white blood cell (WBC) 4.1 × 10^9^/L (3.5–9.5 × 10^9^/L), hemoglobin 73 g/L (130-175 g/L) and platelet count 52 × 10^9^/L (125–350 × 10^9^/L). His SCr level was 5.58 mg/dl, calcium 2.14 mmol/L, serum albumin 34.5 g/L (40–55 g/L) and serum lactate dehydrogenase level 198 IU/L (100–240 IU/L). His urinalysis showed proteinuria 1+ and hematuria (12–15 red blood per high power field). Urine protein excretion was 1.19 g/d with 70.1% of albumin, 25.5% of low molecular weight protein and 4.4% of high molecular weight protein. Urine albumin creatinine ratio (ACR) was 1506.82 mg/gCr (< 30 mg/gCr), N-acetyl-glucosaminidase (NAG) 2 U/L (0–21), and α1-microglobulin 152 mg/L (0-12 mg/L). Kidney ultrasound showed enlarged kidneys (right kidney 13 cm, left kidney 14.1 cm) with increased echogenicity. Serum B-type natriuretic peptide (BNP) was 3359 pg/ml (< 100) and cardiac troponin I (cTnI) 0.05 ng/ml (0–0.03). Electrocardiogram was normal. Echocardiography showed enlarged left ventricle, left atria and right atria, left ventricle showed symmetrical hypertrophy, left ventricular ejection fraction was 65%, and there was severe mitral valve regurgitation and moderate tricuspid valve regurgitation. Systolic blood pressure of pulmonary artery was elevated (56 mmHg). Liver enzymes were normal. Monoclonal IgAλ plus free λ spike was identified in both serum and urine by immunofixation electrophoresis. Serum free light chain test was not done. His serum immunoglobulin (Ig) G level was 3.76 g/L (7.23–16.85 g/L), IgA level was 3.55 g/L (0.69–3.82 g/L), and IgM level was 0.24 g/L (0.63–2.77 g/L). Plasma C3 level was 0.566 (0.60–1.50) g/L, and C4 level was 0.195 (0.12–0.36) g/L. Hepatitis B surface antigen (HBsAg), anti-hepatitis C virus (HCV), antihuman immunodeficiency virus (HIV), Treponema pallidum antibody (TP-Ab), anti-nuclear antibodies, anti-neutrophil cytoplasmic antibodies, anti-glomerular basement membrane antibody and anti-phospholipase A2 receptor (PLA2R) antibodies were all negative.

**Fig. 1:**
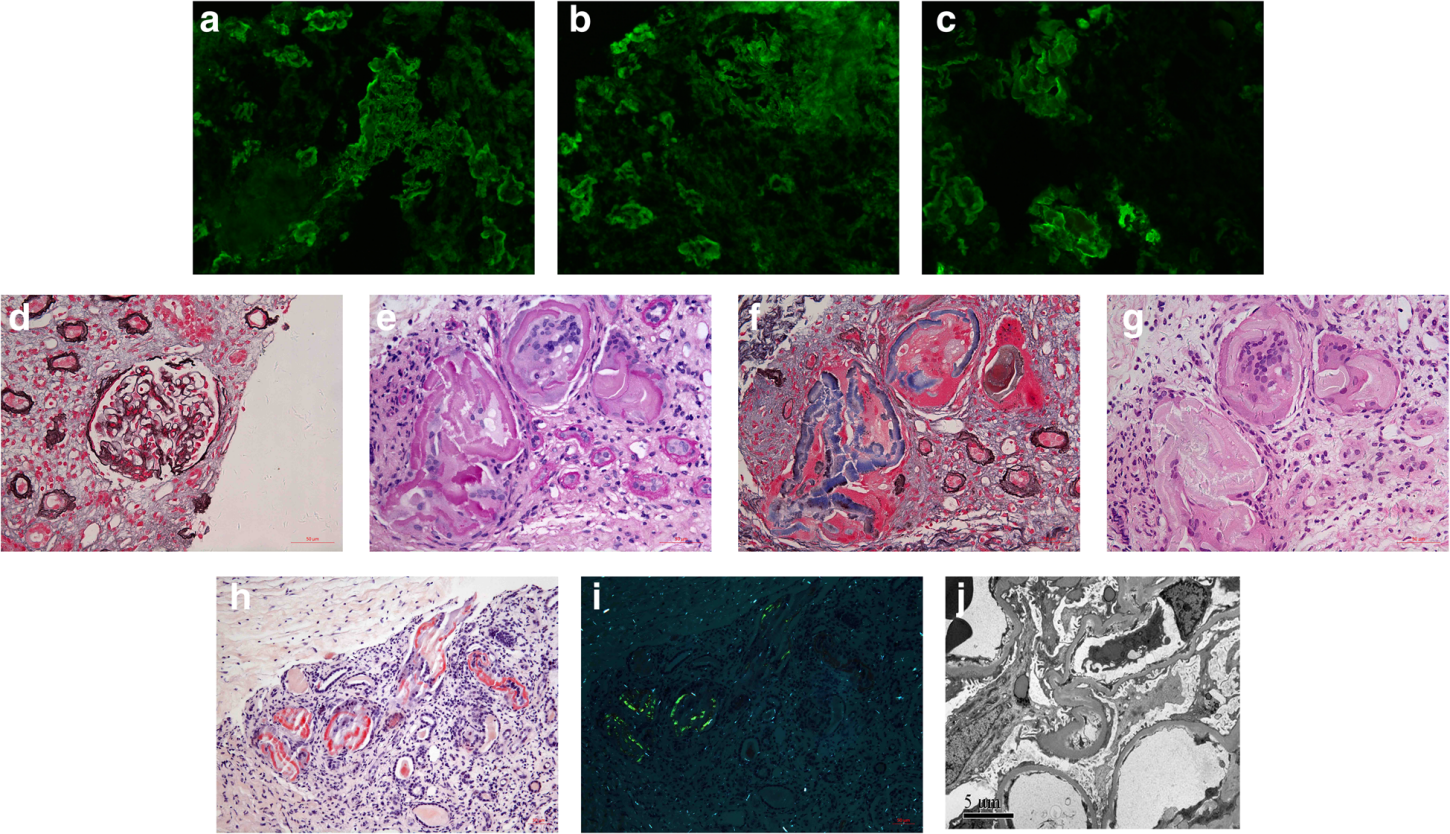
Renal biopsy findings of case 1. **a** showed IgA linear staining along glomerular capillary wall and tubular basement membrane (× 200). **b** showed λ linear staining along glomerular capillary wall and tubular basement membrane (× 200). **c** showed λ was strong positive on the protein casts (× 200). **d** showed minimal mesangial proliferation of the glomeruli (PASM+Masson, × 400). **e** showed PAS negative protein casts in the tubular lumen (PAS, × 400). **f** showed fibrillary structure in the peripheral of the protein casts (PASM+Masson, × 400). **g** showed mononuclear cells in the center of the protein casts (H&E, × 400). **h** showed the protein casts was Congo red positive (Congo red staining, × 200). **i** showed the protein revealed apple-green birefringence with polarized microscopy (Congo red staining, × 200). **j** showed normal glomerular without fine granular deposits along the capillary wall (× 6000)

To identify the cause of albumin-dominant proteinuria and acute kidney disease, the patient underwent renal biopsy on October 16th, 2017. Direct immunofluorescence (IF) examination of frozen renal tissue revealed IgA++ (Fig. 1a), λ++ (Fig. 1b) depositing along the glomerular capillary wall and tubular basement membrane, but not κ. Some intratubular casts showed strong staining for λ+++ (Fig. 1c) with negative κ. No significant deposits for IgG, IgM, C3 and C1q in the glomeruli. Light microscopic examination showed 5 glomeruli. The glomeruli revealed minimal mesangial proliferation (Fig. 1d) with some glomeruli showed ischemic change. Tubular epithelial cells exhibited focal loss of brush border and focal tubular atrophy. Periodic acid-Schiff (PAS) negative thick protein casts were noticed in the tubular lumens (Fig. 1e), the protein casts showed fibrillary structure on silver staining (Fig. 1f). Mononuclear cells were found in the center of the protein casts (Fig. 1g). These protein casts were Congo-red positive (Fig. 1h) and showed apple- green birefringence with polarized microscopy (Fig. 1i). A few protein casts were Congo-red negative. There was very little amyloid deposit in one artery, and no amyloid deposit in glomeruli or interstitia. On electron microscopy, the glomeruli were normal, and there were no deposits along the glomerular basement membrane or tubular basement membrane (Fig. 1j). Only one protein cast was found on electron microscopy but no amyloid fibrils were found on this protein cast.

The final diagnosis of this patient was multiple myeloma (IgAλ) with MCN with diffuse amyloid casts. He was maintained on chemotherapy and hemodialysis. He died 8 months after renal biopsy.

### Case 2

A 58-year-old Chinese man presented with a one-and-a-half-year history of proteinuria. His initial SCr was normal and was treated with tacrolimus and glucocorticoid. However, the patient experienced acute kidney injury with SCr increasing to 1.98 mg/dl afterwards and Tacrolimus was suspended. Then, he switched to oral cyclophosphamide (total 8 g), but reached no remission of proteinuria. To further evaluate the cause of proteinuria, he was admitted to our division in August 2017.

His past medical history and family history was of no significance. On admission, the physical examination revealed a blood pressure of 120/70 mmHg, temperature of 36.5 °C, heart rate of 70/min, and respiratory rate of 18/min. No organomegaly was noticed. Other signs were normal.

After admission, his urinalysis revealed proteinuria 2+ without hematuria. Urine protein excretion was 4.67 ~ 11.117 g/d. His serum albumin 46.9 g/L, SCr level was 1.67 mg/dl, calcium 2.48 mmol/L, uric acid was 510 μmol/L (90–360 μmol/L), serum lactate dehydrogenase level 145 IU/L and cTnI 0.001 ng/ml. Complete blood count showed WBC 4.1 × 10^9^/L, hemoglobin 133 g/L and platelet count 136 × 10^9^/L. Liver enzymes were normal. His serum IgG was 4.33 g/L, IgA was 0.19 g/L, and IgM was 0.13 g/L. Plasma C3 level was 0.873 g/L, and C4 level was 0.411 g/L. Monoclonal IgDλ spike was identified in the serum and urine by immunofixation electrophoresis. Free κ light chain was 8.95 (3.30–19.40) mg/L and free λ light chain was 3775 (5.71–26.30) mg/L. Ultrasonic examination showed enlarged kidneys with increased echogenicity. Electrocardiogram was normal. Echocardiography showed left ventricular ejection fraction was 81.9% and there was mild mitral valve regurgitation and aortic valve regurgitation. Bone marrow aspiration smear revealed 20% of plasma cells. CD38 and CD138 positive cells accounted for 16.6% of bone marrow cells with λ light chain restricted expression as determined by bone marrow flow cytometry. HBsAg, anti-HCV, anti-HIV, TP-Ab, anti-nuclear antibodies, anti-neutrophil cytoplasmic antibodies, anti-glomerular basement membrane antibody and PLA2R antibodies were all negative.

**Fig. 2:**
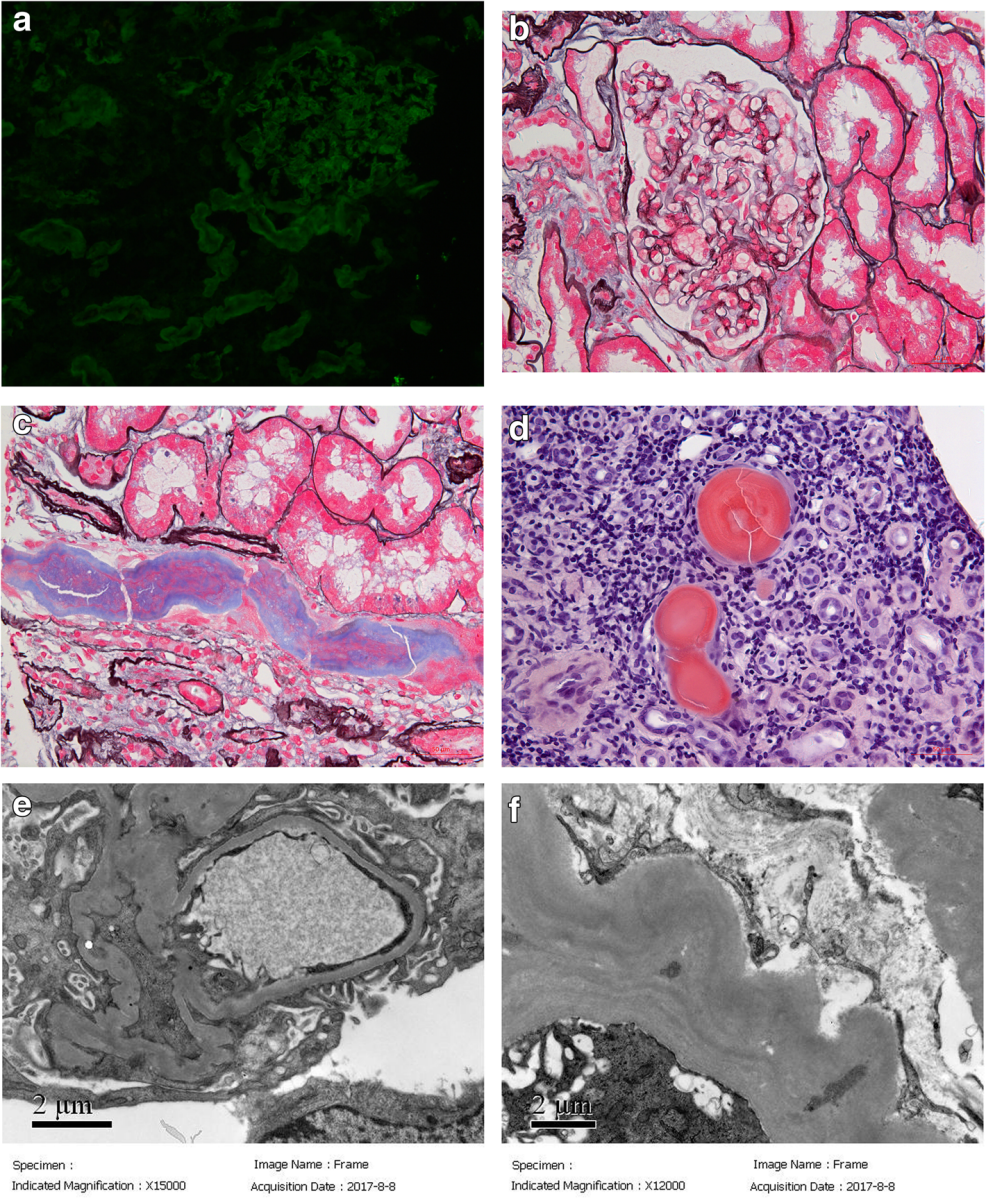
Renal biopsy findings of patient 2. **a** showed λ linear staining along glomerular capillary wall and tubular basement membrane (× 200). **b** showed mild segmental mesangial proliferation of glomeruli (PASM+Masson, × 400). **c** showed fibrillary structure in the peripheral of the protein casts and mononuclear cells in the center (PASM+Masson, × 400). **d** showed the protein cast was Congo-red positive (× 400). **e** showed no deposit in the glomeruli on electron microscopy (× 15,000). **f** showed fine granular deposits along the tubular basement membrane (× 12,000)

To further identify the kidney pathological type, the patient underwent renal biopsy on August 3rd, 2017. Immunofluorescence staining on frozen tissue revealed λ++ linear depositing along the glomerular capillary wall and tubular basement membrane, and κ was negative (Fig. 2a). There are also some intratubular casts showing strong staining for λ++. No significant deposits for IgG, IgA, IgM, C3 and C1q in the glomeruli. Light microscopic examination showed 37 glomeruli and one was globally sclerosed. The glomeruli revealed mild segmental mesangial proliferation (Fig. 2b) and some glomeruli showed ischemic change. Tubular epithelial cells exhibited granular degeneration and focal tubular atrophy. Periodic acid-Schiff (PAS) pale protein casts were noticed in the tubular lumens with fibrillary changes (Fig. 2c), and the protein casts were Congo-red positive (Fig. 2d) showing apple-green birefringence with polarized microscopy. On electron microscopy, the glomeruli basement membrane showed ischemic change without any deposits (Fig. 2e). But there were fine granular deposits along the tubular basement membrane (Fig. 2f).

The final diagnosis of this patient was multiple myeloma (IgDλ) with light chain deposition disease along the tubular basement membrane and MCN with diffuse amyloid casts. The patient was treated with 6 cycles of bortezomib and dexamethasone followed by 3 cycles of lenalidomide, dexamethasone and bortezomib therapy. He achieved very good partial remission (VGPR). Fifteen months after the renal biopsy, his bone marrow plasma cells decreased to 4%. He was on maintaining therapy with IRD (ixazomib, lenalidomide, and dexamethasone) until now. Twenty-seven months after renal biopsy, his SCr was 1.5 mg/dl, and there was no sign of systemic amyloidosis.

## Discussion and conclusions

Multiple myeloma is a hematologic malignant disease with approximately 50% of new-onset MM patients accompanied with renal injury. Renal injury is associated with worse overall survival. The one-year surviving rate was 80% of patients with SCr < 1.5 mg/dl, 50% of those with SCr > 2.3 mg/dl [6] and 30% of those on dialysis [7]. Renal injury in MM may manifest as different pathologic types. Cast nephropathy, light chain amyloidosis and light chain deposition disease are the most common histopathologic lesions [8]. In cast nephropathy, massive glomerular filtered light chains reach the distal tubules and bind with Tamm-Horsfall protein secreted by the thick ascending limb of Henle’s loop. Cast nephropathy usually manifests as acute kidney injury and low molecular light-chain dominant proteinuria. In light chain amyloidosis, unstable free light chains aggregate to form amyloid fibrils and deposit in multiple organs. In kidney, light chain amyloidosis mostly involves the glomeruli and clinically showing albumin-dominant proteinuria. Arteries and arterioles are the second most commonly involved compartment, some patients showing interstitial amyloid deposition and rarely peritubular capillary deposition. Here we reported two rare cases of myeloma cast nephropathy of an unusual variant with amyloid fibrils seen in the casts. No signs of amyloidosis were identified in glomeruli, arteries/arterioles, interstitia and peritubular capillaries, and no evidence of extra-renal amyloidosis. Patient 1 had left ventricular hypertrophy, but it was more likely due to hypertension since the hyperthropy was symmetrical, no sign of low voltage on ECG and serum cTnI was near normal. He did not agree to myocardial biopsy to further exclude cardiac amyloidosis, and gadolinium-enhanced cardiac magnetic resonance was not appropriate due to his decreased renal function.

Histopathologically, these amyloid casts exhibited a peripheral radiating spicular pattern around a central core, which were confirmed using positive Congo-red staining. The casts were weakly eosinophilic, pale periodic acid-Schiff staining, showed blue on trichrome staining and were argyrophilic. They were different from the usual hard myeloma casts with amyloid-like staining properties, including absence of fractures, lack of peri-cast multinucleated giant cells, and lack of the fibrillar ultrastructural appearance on electron microscopy [9]. In the present two cases, most of the protein casts (> 50%) were amyloid casts, and others were usual Conge-red negative protein casts. Among these amyloid casts, the majority of them had a center core of mononuclear cells.

It was not rare to see amyloid casts in MCN cases (27–28%) but those cases were often combined with systemic amyloidosis [10]. There were a few retrospective case studies and case reports (total 26 cases) in the past about MCN with amyloid casts [9–15]. Twenty-five patients (96%) were MM with mostly λ light chain (73%). In most cases (68%), patients showed rapid deterioration of renal functions and 41% patients were on dialysis at the time of renal biopsy. In the MCN cases with systemic amyloidosis data, 87% patients also had extra-renal amyloidosis. Isolated amyloid in protein casts in MM were rare (2 autopsies, and 3 biopsies including our two cases), and these patients should be categorized as MCN with diffuse amyloid deposits (Table 1). After 8 to 27 months follow-up, these 3 biopsy-proven cases of MCN with diffuse amyloid casts patients did not develop any sign of systemic amyloidosis.

**Table 1:** A summary of literature review of MCN with amyloid casts

Journals	Case No	Hematology	κ:λ	Mean age (range)	Sex (F:M)	Amyloid casts (%)	Amyloid deposit in other kidney compartments	Systemic amyloid	Acute kidney injury (n)	Dialysis at diagnosis	Duration of follow up (months)	Endpoint
Virchows Arch A 1980	Autopsy *N* = 4	MM (*n* = 4)	3:1	71 (59–82)	3:1	ND	None	2/4 pancreas	ND	NA	NA	NA
Kidney Int 2007	Biopsy *N* = 1	MM (*n* = 1)	0:1	52	0:1	ND	Vessels and glomeruli	marrow and synovia	1	1	NA	ESRD, alive
Kidney Int 2008	Biopsy *N* = 1	MM (*n* = 1)	0:1	60	0:1	ND	None	ND	1	1	NA	ESRD, alive
Am J Kidney Dis 2009	Biopsy *N* = 1	MM (*n* = 1)	0:1	59	1:0	ND	None	ND	1	1	NA	ESRD, alive
Intern Med 2015	Biopsy *N* = 1	MM (*n* = 1)	0:1	74	0:1	ND	None	ND	0	1	15	ESRD, alive
Mod Pathol 2018	Biopsy *N* = 17	MM (*n* = 16)	4:13	67 (47–87)	7:10	< 5%: *n* = 9 5% ~ 25%: *n* = 3 > 25%:*n* = 5	4 in tubular cells	17/17	11	4	64 (Median)	Median OS: 21 months
Kidney Int 2019	Biopsy *N* = 1	MM (*n* = 1)	0:1	53	1:0	ND	None	None	1	1	22	ESRD, alive

Abbreviations

*NA* not applicable

*ND* not determined

*OS* overall survival

**Fig. 3:**
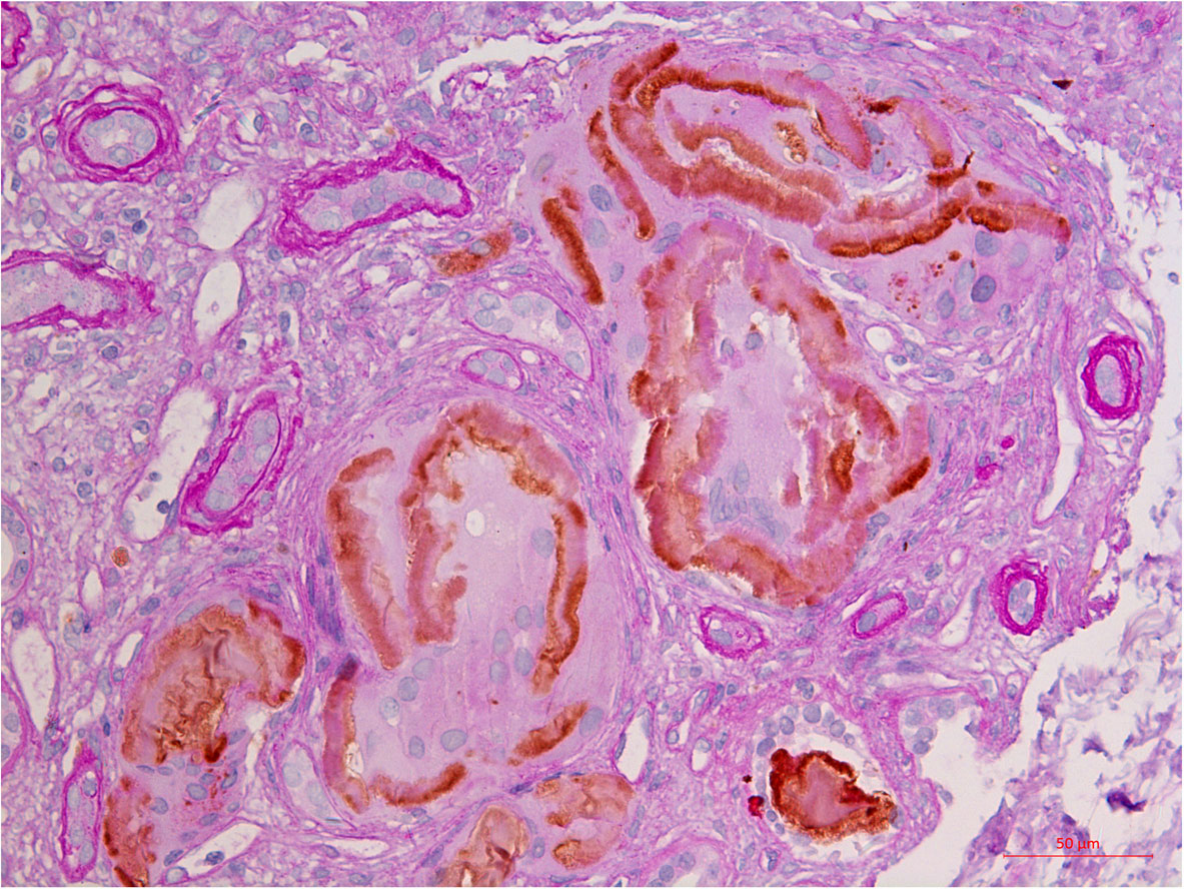
Immunohistochemistry staining of CD68 of patient 1. The mononuclear cells in the center of the amyloid casts were CD 68 negative. (CD68 + PAS, × 400)

The underlying mechanisms of those amyloid casts are unclear. MCN with diffuse amyloid casts had no other renal and extra-renal amyloidosis, suggesting local factors in the pathogenesis of amyloid formation. Using a well-established animal model, it has been demonstrated that mesangial cells could process abnormal monoclonal free light chain to form amyloid fibrils [16]. Notably, in our two cases, CD68 negative mononuclear cells were identified in the peripheral or in the center of most amyloid casts (Fig. 3). Based on the morphology and location, it was reasonable to assume that the mononuclear cells in the center of amyloid casts might be tubular epithelial cells. Similar to the mechanism of mesangial cells processing free light chains to form amyloid fibrils, it was possible that, in MCN with diffuse amyloid casts, the proximal tubular epithelial cells absorbed the free light chains with special biochemical characteristics via cubulin-meglin complex. The free light chains were processed in the endosome-lysosome system and cannot be fully digested. The undigested free light chain fragments were then secreted or desquamated into the tubular lumen and form amyloid fibrils under certain environment. Further well-designed studies are needed to explore the underlying pathogenesis of MCN with diffuse amyloid casts.

In conclusion, we reported 2 cases of MCN with diffuse amyloid casts. These amyloid casts have different histopathologic characteristics from the usual myeloma casts. The special biochemical characteristics of free light chain and tubular epithelial cells might play important roles in the pathogenesis of MCN with diffuse amyloid casts.

## Data Availability

All data generated or analyzed during this study are included in this published article.

## Abbreviations

ACR: Albumin creatinine ratio
BNP: Serum B-type natriuretic peptide
C1q: Component C1q
C3: Complement 3
C4: Complement 4
cTnI: Cardiac troponin I
HBsAg: Hepatitis B surface antigen
HCV: Hepatitis C virus
HIV: Human immunodeficiency virus
IgA: Immunoglobulin A
IgD: Immunoglobulin D
IgG: Immunoglobulin G
IgM: Immunoglobulin M
IRD: Ixazomib, lenalidomide, dexamethasone
LCPT: Light chain proximal tubulopathy
MCN: Myeloma cast nephropathy
MIDD: Monoclonal Ig deposition disease
MM: Multiple myeloma
NAG: N-acetyl-glucosaminidase
PAS: Periodic acid-Schiff
PCD: Bortezomib, cyclophosphamide and dexamethasone
PD: Bortezomib and dexamethasone
PDDT: Bortezomib, doxorubicin, dexamethasone and thalidomide
PLA2R: Phospholipase A2 receptor
PTD: Bortezomib, thalidomide and dexamethasone
SCr: Serum creatinine
TCD: Thalidomide, cyclophosphamide and dexamethasone
TD: Thalidomide and dexamethasone
TIN: Tubulointerstitial nephritis
TP-Ab: Treponema pallidum antibody
VGPR: Very good partial remission
